# Fine Root Productivity and Turnover of Ectomycorrhizal and Arbuscular Mycorrhizal Tree Species in a Temperate Broad-Leaved Mixed Forest

**DOI:** 10.3389/fpls.2016.01233

**Published:** 2016-08-26

**Authors:** Petra Kubisch, Dietrich Hertel, Christoph Leuschner

**Affiliations:** Plant Ecology and Ecosystems Research, Albrecht von Haller Institute for Plant Sciences, University of GöttingenGöttingen, Germany

**Keywords:** *Acer* Carpinus, Fagus, Fraxinus, root branching order, *Tilia*

## Abstract

Advancing our understanding of tree fine root dynamics is of high importance for tree physiology and forest biogeochemistry. In temperate broad-leaved forests, ectomycorrhizal (EM) and arbuscular mycorrhizal (AM) tree species often are coexisting. It is not known whether EM and AM trees differ systematically in fine root dynamics and belowground resource foraging strategies. We measured fine root productivity (FRP) and fine root turnover (and its inverse, root longevity) of three EM and three AM broad-leaved tree species in a natural cool-temperate mixed forest using ingrowth cores and combined the productivity data with data on root biomass per root orders. FRP and root turnover were related to root morphological traits and aboveground productivity. FRP differed up to twofold among the six coexisting species with larger species differences in lower horizons than in the topsoil. Root turnover varied up to fivefold among the species with lowest values in *Acer pseudoplatanus* and highest in its congener *Acer platanoides*. Variation in root turnover was larger within the two groups than between EM and AM species. We conclude that the main determinant of FRP and turnover in this mixed forest is species identity, while the influence of mycorrhiza type seems to be less important.

## Introduction

Leaves and fine roots are the organs that supply the plant with energy, water and nutrients. Because of their paramount importance for life, trees invest a large part of their annual carbon gain into the formation of new leaves (∼30%) and fine roots (∼20–40% or more, Keyes and Grier, 1981; Vogt et al., 1996; Müller-Haubold et al., 2013). While the annual production of leaf mass and the phenology of leaf formation and abscission are easily measured in temperate deciduous trees, it is much more difficult to investigate the production and turnover of fine roots (conventionally defined as roots <2 mm in diameter). This is due to the inconspicuous life of roots in the soil, but also because fine roots are not shed synchronously as defined entities at the end of their life like leaves. Rather, fine root death occurs gradually in the more distal root segments (Xia et al., 2010) and new first-, second-, and higher-order root segments produced during a subsequent flush of root growth may replace the shed root segments (Fitter, 1996). Thus, the most distal root segments of lowest root order generally are more short-lived than more proximate higher-order segments, and root turnover (and its inverse, root lifespan) varies across the fine root system, in marked contrast to foliage (Withington et al., 2006).

Understanding the factors that influence fine root lifespan is important because root growth consumes a substantial amount of the annually produced carbohydrates, thereby lowering timber production (Fogel, 1983; Hertel et al., 2013). Moreover, root litter represents an important, if not the largest, source of carbon in forest soils (Fogel, 1983; Rumpel et al., 2002; Fan and Guo, 2010). Most studies on the fine root dynamics of temperate tree species were conducted with juvenile plants in common garden experiments without interspecific root system interactions (e.g., Withington et al., 2006; McCormack et al., 2014). An alternative approach is the comparison of different forest types (e.g., Guo et al., 2008b; Brunner et al., 2013), where species differences in fine root dynamics may be confounded by different site conditions. A few studies have investigated fine root lifespan and productivity in mature mixed forests (e.g., Tierney and Fahey, 2001; Meinen et al., 2009a,b), but these studies did not attempt to explain species differences in root dynamics. Eissenstat et al. (2015) were the first to relate fine root productivity (FRP) and lifespan in a mixed forest to the root morphologies and foraging strategies of the different co-occurring tree species, comparing six arbuscular mycorrhizal (AM) species of the genera *Magnolia, Liriodendron, Juglans, Fraxinus, Acer*, and *Ulmus*.

In western Eurasian cool-temperate broad-leaved forests, the majority of tree species are forming ectomycorrhizae (EM) as do, for example, species of the genera *Fagus, Quercus, Tilia, Carpinus*, and *Betula*. A few AM species (genera *Acer, Fraxinus, Prunus*, and *Ulmus*) co-occur with the dominant EM species in these forests. It is not known whether the two main types of mycorrhizal symbiosis are linked to contrasting fine root traits in terms of root lifespan and growth rate, when the species are co-occurring in the same stand. Different fine root dynamic properties of EM and AM tree species, if existing, could reflect different strategies of belowground resource foraging, given that EM species are thought to be more efficient in terms of nitrogen acquisition and AM species of phosphorus acquisition. Such differences might also explain why EM tree species dominate cool-temperate and boreal forests and AM species are much more abundant in tropical and sub-tropical forests (McGuire et al., 2008; Lang et al., 2011).

In this study, we examined the FRP of each three co-occurring EM and AM tree species in a natural temperate broadleaf mixed forest employing a modified ingrowth core technique according to Meinen et al. (2009b) and Hertel et al. (2013) combined with root coring for biomass determination. This allowed calculating fine root turnover in the <2 mm-diameter class and obtaining an estimate of the average lifespan of the root mass in this fraction. The six species (*Fraxinus excelsior, Acer pseudoplatanus, Acer platanoides, Carpinus betulus, Tilia cordata*, and *Fagus sylvatica*) are abundant tree species in central European woodlands and highly (or moderately) important for forestry. They differ not only with respect to mycorrhiza type, but also in terms of canopy architecture, shade tolerance, hydraulic architecture, and their role in forest succession (Köcher et al., 2009; Ellenberg and Leuschner, 2010; Legner et al., 2014). Moreover, fine root morphology differs not only between the genera but also between the closely related *Acer* species (Meinen et al., 2009a; Jacob et al., 2012; Kubisch et al., 2015). The species *A. pseudoplatanus, A. platanoides, T. cordata*, and *F. sylvatica* were also studied by Withington et al. (2006) in a common garden experiment, which allows comparison of results, even though tree age, stand density and also methodology (ingrowth core vs. mini-rhizotron approach) differed between the two studies. By investigating the six species’ fine root turnover in the same mixed forest in patches with contrasting species dominance, we were able to compare mature trees under natural growing conditions on similar soil. This study builds on an earlier investigation of fine root morphological traits of these species, which showed that root morphology depended mainly on species identity, while mycorrhiza type was of secondary importance (Kubisch et al., 2015). In the present study, we focus on FRP and root lifespan of the six tree species, testing the hypotheses that (i) coexisting AM and EM tree species differ in fine root turnover and root productivity, reflecting different nutrient acquisition strategies, (ii) FRP increases with decreasing mean fine root diameter of the species (Eissenstat, 1991), and (iii) FRP is higher, and root lifespan shorter, in tree species with higher aboveground productivity. The latter assumption relates to the generally higher nutrient and water demand of fast-growing species, which might be associated with thinner, more short-lived fine roots (Eissenstat et al., 2015). By comparing the two maple species *A. pseudoplatanus* and *A. platanoides*, we further tested for congeneric contrasts in fine root dynamics in two closely related tree species.

## Materials and Methods

### Study Site and Plot Design

The study was conducted in Hainich National Park in Thuringia, central Germany, which protects one of the largest remaining temperate deciduous broadleaf forests in Central Europe (7500 ha). Beside large areas of monospecific European beech (*F. sylvatica* L.) forest, the park contains forest stands with relatively high tree species richness. During the past 50 years, this forest was exposed to only minor management activities in form of selective logging. With declaration of a national park in 1997, all activities like logging and military training, practiced in certain areas, were abandoned.

The study site is located on a Triassic limestone plateau (Muschelkalk formation; 308–399 m a.s.l.; 51°04′ N, 10°30′ E) within the ‘Thiemsburg area’ in the north-east of the national park, where more than six tree species co-occur either in quasi-random mixture or in small groups consisting of 3–6 trees of a species. The species in this study, i.e., European beech (*F. sylvatica*), Small-leaved lime (*T. cordata* Mill.), European hornbeam (*C. betulus* L.), European ash (*F. excelsior* L.), Sycamore maple (*A. pseudoplatanus* L.) and Norway maple (*A. platanoides* L.), were the species with highest abundance in this Stellario-Carpinetum community (oak-hornbeam forest). Three of the six species are ectomycorrhizal (EM; *C. betulus*, *F. sylvatica*, and *T. cordata*), while the other species were found to form only AM in the study site (*A. pseudoplatanus, A. platanoides*, and *F. excelsior*); colonization by both AM- and EM-forming fungi as found in some *Acer* species (e.g., Smith and Read, 2007) was not observed (see also Meinen et al., 2009a,b; Jacob et al., 2010). Mean annual precipitation in the study region is ∼ 590 mm year^-1^ and mean annual temperature 7.5°C (periods 1973–2004, Deutscher Wetterdienst 2005). In the study years 2012 and 2013, mean annual air temperatures of 9.7°C (2012) and 8.5°C (2013) and precipitation totals of 603 mm (2012) and 598 mm (2013) were recorded at the nearest weather station Weberstedt/Hainich (Deutscher Wetterdienst, 2009).

The soil of the study area is a base-rich Eutric Luvisol (FAO taxonomy 2006) with a profile depth of 60–70 cm developed in clay-rich Pleistocene loess that covers the limestone bedrock. Due to high clay content, the soil can dry out strongly in summer, while it may show partly stagnant properties during spring and winter. The plots were established in patches with dominance of each one of the six species which were characterized by similar soil properties. Marginal differences in soil conditions between the plots of the species were primarily caused by the specific litter properties of the species (Rothe and Binkley, 2001; Guckland et al., 2009). For example, the base saturation at the cation exchangers in the topsoil and lower mineral soil was marginally lower under *Fagus* (78.5%) than under the other species (Kubisch et al., 2015; **Table 1**). However, none of the measured properties differed significantly between the plots.

**Table 1:** Aboveground structural characteristic of the target trees and of entire study plots; all species in a plot for the six plot types (species); important soil chemical properties of the mineral topsoil (0–10 cm) are also indicated.

Variable	Species					
	*F. excelsior*	*A. pseudoplatanus*	*A. platanoides*	*C. betulus*	*T. cordata*	*F. sylvatica*
**Target species**						
Tree height (m)	32.3 ± 1.5	28.6 ± 0.9	23.8 ± 2.0	22.8 ± 1.1	24.2 ± 1.40	26.4 ± 0.7
AWB (Mg ha^-1^)	313.2 ± 47.1	182.8 ± 30.7	136.1 ± 21.8	168.7 ± 34.6	169.1 ± 24.9	207.0 ± 24.2
AWB (Mg Ind. tree^-1^)	1.9 ± 0.3	1.8 ± 0.1	1.4 ± 0.2	1.1 ± 0.2	0.8 ± 0.1	1.2 ± 0.1
Dbh (cm)	52.2 ± 3.5	58.1 ± 3.2	51.2 ± 3.6	43.4 ± 3.3	46.4 ± 2.3	43.5 ± 2.2
BA (m^2^ ha^-1^)	47.82 ± 4.69	27 ± 4.01	22.07 ± 3.24	26.29 ± 5.16	43.60 ± 6.12	52.32 ± 5.04
**All species per plot**						
Total BA (m^2^ ha^-1^)	57.1 ± 5.4	47.8 ± 8.8	28.7 ± 2.5	31.6 ± 6.6	50.9 ± 6.0	60.3 ± 7.8
Proportion of target species (%)^1^	83.6 ± 3.5	62.7 ± 7.2	77.4 ± 9.9	86.1 ± 8.0	84.8 ± 4.0	90.4 ± 5.7
Stem density (no. trees ha^-1^)	409 ± 71	376 ± 64	232 ± 23	232 ± 41	365 ± 35	696 ± 98
**Soil chemical properties (0–10 cm)**					
C/N g g^-1^	11.9 ± 0.2	11.6 ± 0.4	12.0 ± 0.4	12.5 ± 0.2	12.0 ± 0.3	12.6 ± 0.3
CEC (μmol_c_/g dry soil)	213.5 ± 75.5	195.8 ± 69.2	206.5 ± 73.0	178.5 ± 63.1	192.2 ± 67.9	139.2 ± 49.2
Base saturation (%)	91.2 ± 5.8	88.8 ± 4.8	87.3 ± 5.4	88.0 ± 6.8	93.4 ± 4.2	78.5 ± 8.2
pH (H_2_O/KCl)	5.29/4.19	5.07/4.10	5.20/4.11	5.29/4.15	5.41/4.31	5.02/3.94

*Given are mean ± SE for *n* = 8 plots. Aboveground woody biomass (AWB) is given for spring 2012. BA, basal area of the target species; CEC, cation exchange capacity (data taken from Kubisch et al., 2015; see Materials and Methods, contact this source). ^1^% of BA*.

Circular plots (diameter 12 m; area 113 m^2^) containing mature trees of one of the six target species (‘tree clusters’) were randomly selected in the area. For our analysis, two neighboring trees of the target species with dominant position in the upper canopy layer, or one dominant tree of the respective species, were chosen; they formed the center of the plots. The selected trees had diameters at breast height (dbh) of 40–60 cm (**Table 1**). This plot scheme was chosen to minimize possible species effects on soil chemistry in the mixed forest, which would have been more pronounced in larger monospecific stands. The scheme ensures that the bulk of fine roots in the soil belonged to the target species (typically >80%). We sampled eight plots per species resulting in 48 plots in total. All stems >10 cm dbh in a ‘tree cluster’ were investigated for their species identity, dbh, basal area and tree height (**Table 1**).

### Fine Root Productivity and Root Dynamics

For quantifying FRP (in g m^-2^ year^-1^), we applied a modified ingrowth core approach. To achieve more natural root growth conditions in the cores, we modified the conventional ingrowth core technique (Persson, 1980; Powell and Day, 1991; Majdi, 1996) and refrained from enclosing the core in a net in order to minimize soil disturbance. Compared to other techniques, the ingrowth core method has been found to produce rather conservative figures of FRP in temperate forests (e.g., Hertel and Leuschner, 2002; Hendricks et al., 2006). The ingrowth cores were installed in June 2011 immediately after an inventory of standing fine root biomass (FRB; diameter <2 mm) which was conducted in the same 48 plots by coring the topsoil to 30 cm depth at 150 cm distance to the stem of the central tree in the plot using a steel corer of 35 mm diameter (Kubisch et al., 2015). The sampling holes were refilled with root-free soil from a nearby place (distance ca. 30–50 cm) and used as ingrowth cores for a period of 2 years. Each refilled coring site was precisely marked with three plastic sticks inserted down to 30 cm soil depth which allowed a resampling of the core at exactly the same place after 24 months. Earlier investigations at the same forest sites had shown that re-colonization of the cores by fine roots started typically 12 months after their installation (Meinen et al., 2009b). We thus assumed that fine root growth in the cores took place from May 2012 to May 2013, i.e., over 365 days, while the period of core exposure lasted from July 1, 2011 to May 16, 2013. The cores were resampled on May 16, 2013 in the same manner as done in the initial biomass inventory in 2011 (Kubisch et al., 2015). Upon harvest, the extracted soil cores were divided into the 0–10, 10–20, and 20–30 cm soil layers and stored in plastic bags at 4°C. The root samples were subsequently analyzed in the lab within 3 months by carefully rinsing the soil cores with tap water over a sieve of 0.25 mm mesh size and extracting all fine root branches >10 mm length. Assignment of root mass to tree species was done with a morphological key that has been developed earlier in this stand using periderm properties such as color and surface structure, the mode of root branching, and mycorrhiza type as criteria (Hölscher et al., 2002; Meinen et al., 2009a; Kubisch et al., 2015). Properties like elasticity of the stele, and the cohesion of periderm and stele were used for distinguishing live from dead root mass (Persson, 1978; Hertel and Leuschner, 2002). The turnover of fine root mass (unit: year^-1^; i.e., the inverse of root longevity) was calculated by dividing annual FRP by standing FRB (inventory data). Turnover data are bulk values for the entire fine root biomass <2 mm in diameter, thus averaging root lifespan over all root orders. At the date of harvest, no fine root necromass was observed in the ingrowth cores and this component was thus not considered in the analysis of FRP and turnover.

All extracted live fine roots from the ingrowth cores were scanned and analyzed for their specific surface area (SRA, in cm^2^ g^-1^), specific length (SRL, in m g^-1^), and tissue density (RTD, in g cm^-3^) using a flat-bed scanner and WinRhizo software (Régent Instruments, Inc., Québec City, QC, Canada). The annual production of fine root length (m per m^2^ ground area and year) and fine root surface area (m^2^ m^-2^ year^-1^) was calculated using the measured morphological characteristics of the fine roots collected in the ingrowth cores.

**FIGURE 1:**
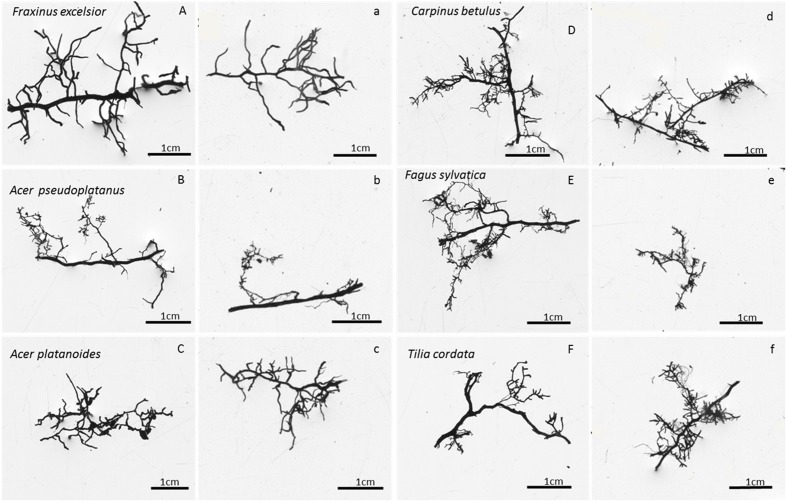
**Photographs of typical terminal fine root branches of the six species **(A–F)** as collected in soil cores of the inventory (respective left columns, marked with capital letters) or in the ingrowth cores (respective right columns, marked with small letters)**. Images were taken with WinRhizo software.

To obtain a rough estimate of the fine root biomass produced annually in the different root orders, we used the percent distribution of fine root biomass to the root orders #1 to #4 which was determined in a detailed root order analysis of the standing FRB inventory done by Pregitzer et al. (2002), Kubisch et al. (2015). This analysis was conducted in a representative sub-sample of the FRB material of every soil sample and soil depth (>10 mm length) which had been subjected to a fractionation into root orders. The individual segments of a root branch were assigned to the first four branching orders according to the ordering system proposed by Fitter (1987) and Pregitzer et al. (2002) and dissected into the orders using a razorblade. For all species except *F. excelsior*, the root tip(s) was counted together with the adjacent root segment as first-order segment, as it was often not possible to clearly recognize the transition between tip and subsequent root segment. The separation was especially problematic in case of the EM tree species which often formed coralloid cluster-like structures that could not be split into first and second order segments (Valtanen et al., 2014). In contrast, in *F. excelsior* with AM, the individual tips were clearly recognizable and hence were counted as first root order. Root segments of the fifth or higher orders contributed with less than 5% to the fine root biomass <2 mm and were lacking in various samples; they were not considered in the subsequent analysis. The proportion of the first to fourth root orders in the total fine root mass of a root branch was detected and the ratios were applied to estimate the production of root mass in the four orders in the ingrowth cores. This extrapolation can give only a very rough number as it is based on the assumption that fine roots growing into the ingrowth cores do not differ in their branching structure from the fine roots collected in the inventory (**Figure 1**), and root longevity is similar in the root orders. The latter assumption is probably not valid (McCormack et al., 2015) suggesting that our calculation can only indicate the magnitude of root biomass produced in the different orders. We thus use these data only as an estimate of the production of absorptive roots (first- and second-order roots) per ground area of the six species.

After the detailed analysis, all extracted fine root material was dried at 70°C for 48 h until constant weight. The carbon and nitrogen concentrations were analyzed by gas chromatography (vario EL, elementar, Hanau, Germany) in the ground root material of the initial FRB inventory.

### Aboveground Woody Biomass Production

The annual production of above-ground woody biomass (ABWP in Mg ha^-1^ year^-1^; including stem and larger branches) was calculated from stem increment data obtained for every target tree using permanently installed dendrometer tapes (UMS, München, Germany) mounted at 1.50 m stem height. The height of the target trees was measured at the beginning of the study using a Vertex IV ultrasonic height meter (Haglöf, Langsele, Sweden). To calculate the total aboveground woody biomass (AWB) of the trees, we used the following allometric equations given for the respective species in Zianis et al. (2005):

*F. sylvatica* and *C. betulus*:

$$A\,W\,B=0.04736\cdot d\,b\,h^{1.80521}\cdot h^{0.99603}\,\;\;\;\;\;\;\;\;\;\;\;\;\;\;\;\;\;\;\;\;\;\;\;\;\;\;\left(1\right)$$

*T. cordata*:

I⁢n⁢(A⁢W⁢B)=−2.6788+2.4542⋅I⁢n⁢(d⁢b⁢h)⁢                           (2)

*A. pseudoplatanus and A. platanoides*:

I⁢n⁢(A⁢W⁢B)=−2.7606+2.5189⋅I⁢n⁢(d⁢b⁢h)⁢                           (3)

*F. excelsior*:

I⁢n⁢(A⁢W⁢B)=−2.4598+2.4882⋅I⁢n⁢(d⁢b⁢h),                           (4)

with AWB being total aboveground woody biomass including branches (in kg per tree), dbh diameter at breast height (in cm), and h tree height (in m).

For *C. betulus*, no specific allometric equation was found in the literature. We thus used the equation for *F. sylvatica* as an approximation. For *A. platanoides*, we used the same equation as for *A. pseudoplatanus* because specific equations seem to lack for this species as well. We assumed that height growth during the relatively short (1 year) measuring period was negligible and excluded it from the calculations. Annual woody biomass production (ABWP) of the target trees in the plots was then calculated as the change in aboveground woody biomass of each tree from spring 2012 to spring 2013.

### Statistical Analyses

All data sets were tested for normal distribution using a Shapiro–Wilk test. As normal distribution was often not given and the data sets could not sufficiently be transformed, non-parametric statistics were applied in these cases. For most variables, a Kruskal–Wallis *H*-test followed by a Mann–Whitney *U*-test for pairwise comparison between the species was conducted (*p* < 0.05). The relationship between ABWP, or root morphological traits, and the FRP or root turnover of the six species was explored with Spearman rank correlation analysis. ANCOVA was employed to separate between effects of mycorrhiza type and effects of various root morphological traits on fine root turnover. These tests were conducted with SAS 9.3 software. In order to analyze the inter-relationships between fine root biomass, root morphological properties, FRP, root turnover, and aboveground tree structure, biomass and wood production, we conducted a principal components analysis (PCA) using the package CANOCO, version 4.5 (Biometris, Wageningen, The Netherlands).

## Results

### Fine Root Productivity and Turnover

**FIGURE 2:**
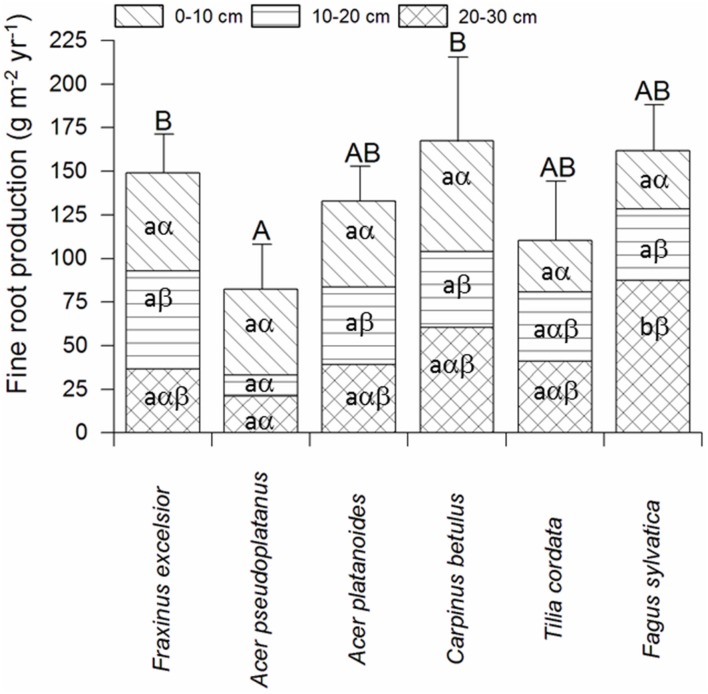
**Fine root productivity (FRP) of the six tree species in the three soil depths according to the ingrowth core study (mean ± SE; *n* = 8 plots)**. Different capital letters indicate significant differences (*p* < 0.05) between the species in the soil profile (0–30 cm); significant differences between the soil depths for a given species are indicated by different lower case Latin letters, differences between tree species within a given soil depth by lower case Greek letters.

**FIGURE 3:**
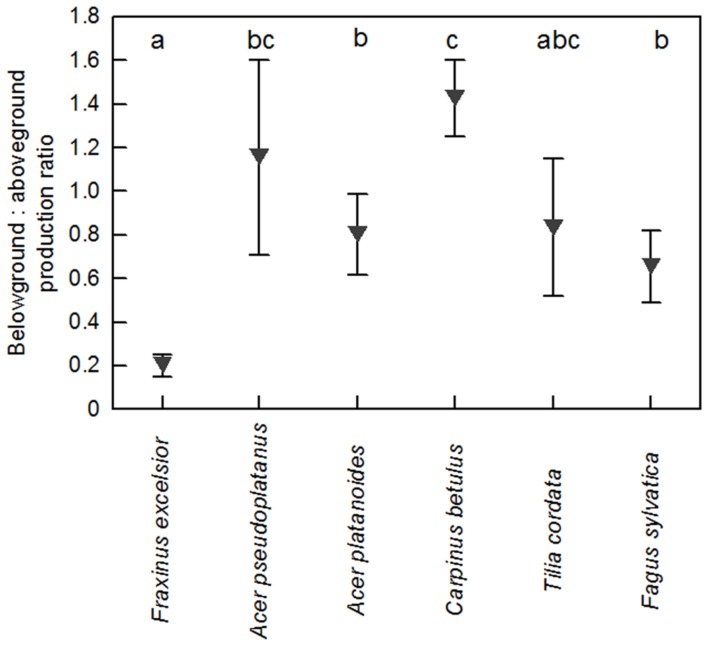
**Ratio of annual belowground (fine root) to aboveground (woody biomass) production in the six species**. FRP was expressed per m^2^ ground area; woody biomass production is the growth of the target trees. Statistically significant differences between the species are indicated by different letters.

Fine root biomass per ground area (0–30 cm profile) ranged between 140 and 300 g m^-2^ among the six tree species in the plots with dominance of the respective species (Supplementary Table SI1). As for biomass, annual FRP in the soil profile varied by a factor up to two among the species. Highest productivity was measured for *C. betulus, F. sylvatica*, and *F. excelsior* (∼150–170 g m^-2^ year^-1^), intermediate values for *A. platanoides* and *T. cordata*, and lowest for *A. pseudoplatanus* (∼80 g m^-2^ year^-1^, **Figure 2**). Interestingly, the six species differed not significantly in FRP in the uppermost soil layer (0–10 cm), while marked differences existed in the two deeper soil layers. Particularly high productivity was measured in the 20–30 and 10–20 cm layers for *F. sylvatica*, while *A. pseudoplatanus* reached only low values in these depths (**Figure 2**). When FRP was related to aboveground woody biomass production [ABWP, measured as kg biomass increment per target tree(s) per m^2^], *C. betulus* and *A. pseudoplatanus* reached highest ratios (>1), *T. cordata, A. platanoides*, and *F. sylvatica* intermediate ratios (0.6–0.8), and *F. excelsior* a very low ratio (<0.2; **Figure 3**).

**FIGURE 4:**
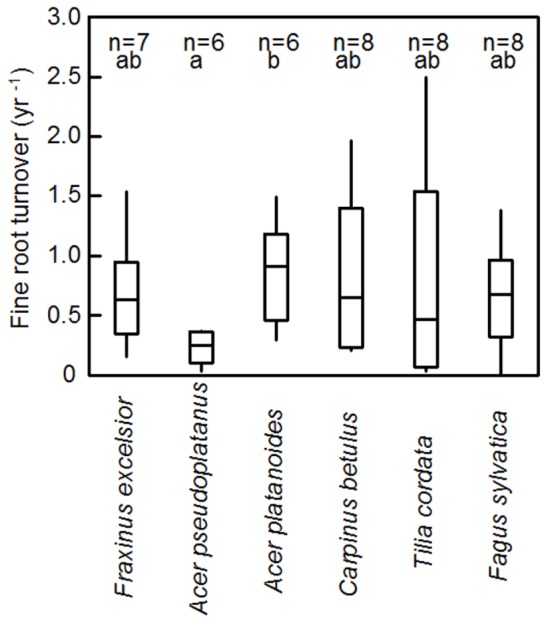
**Median fine root turnover (year^-1^) of the six tree species according to ingrowth core data for the 0–30 cm profile**. Given are the median, the 25- and 75- percentiles and the minima and maxima. Significant differences (*p* < 0.05) between the species are indicated by different letters.

Fine root turnover (productivity per standing biomass) in the 0–30 cm profile was highest in *A. platanoides* and lowest in *A. pseudoplatanus* (difference significant; **Figure 4**). However, the variation in turnover values within a species was generally large (ranging from 0.1 or 0.2 to 2.0 year^-1^ or higher). Consequently, in most cases, species differences in root turnover were not significant. Moreover, there was no uniform trend of fine root turnover change with soil depth across the six species (**Table 2**). *C. betulus* and *T. cordata* showed a decrease in turnover with depth, while *F. sylvatica*, *F. excelsior* and the two *Acer* species had the highest fine root turnover in the deepest (20–30 cm) layer (**Table 2**). However, the variation in turnover figures within a species and soil depth was also large. Neither fine root turnover nor FRP differed significantly between the EM and AM species groups (**Table 3**). Analysis of covariance with mycorrhiza type as independent and fine root turnover as dependent variable revealed that among several root morphological traits introduced as covariates, only SRL had a significant influence (*p* = 0.008; results not shown).

**Table 2:** Median of fine root turnover (year^-1^) of the six species in the three different soil depths.

	Soil depth		
	0–10 cm	10–20 cm	20–30 cm
*Fraxinus excelsior*	0.42 aAB	0.16 aAB	0.44 aA
*Acer pseudoplatanus*	0.22 aA	0.21 aAB	0.44 aA
*Acer platanoides*	1.14 aB	1.18 aA	1.60 aB
*Carpinus betulus*	0.70 aAB	0.60 aAB	0.22 aA
*Tilia cordata*	0.50 aAB	0.17 aAB	0.36 aA
*Fagus sylvatica*	0.42 aAB	0.33 aB	0.70 aAB

*Significant differences (pairwise comparison; Mann–Whitney *U*-test; *p* < 0.05) between the soil depths for a species are marked with different lower case letters, those for two species at a given soil depth are marked with different capital letters*.

**Table 3:** Comparison between the EM and AM tree species in terms of fine root turnover and fine root productivity (FRP).

Mycorrhiza type	No. of species	Turnover (year^-1^)	FRP (g m^-2^ year^-1^)
AM	3	0.72 ± 0.22	121.51 ± 20.07
EM	3	0.60 ± 0.07	146.49 ± 18.15

*Given are the group mean ± SE, based on the species’ median turnover and mean FRP. Both differences were not significant at *p* < 0.05 according to a Mann–Whitney *U*-test*.

### Annual Production of Fine Root Length and Surface Area

Fine root length growth in the 0–30 cm soil profile ranged from 1359 m m^-2^ year^-1^ in *T. cordata* to 3303 m m^-2^ year^-1^ in *F. excelsior* (difference significant between the low values in *T. cordata* and *A. pseudoplatanus*, and the high values in *F. excelsior* and *C. betulus*). Most species had the highest fine root elongation rate in the upper 10 cm and a decrease to lower layers (**Table 4**). However, *T. cordata* and *A. platanoides* had the highest fine root length production in 10–20 cm depth and *F. sylvatica* showed a significant increase in fine root length production from 0–10 to 20–30 cm depth (**Table 4**). As expected, annual fine root surface area production revealed a similar pattern as length production; *F. excelsior* produced the largest root surface area per year (4.1 m^2^ m^-2^ year^-1^), *T. cordata* the lowest (1.7 m^2^ m^-2^ year^-1^). Fine root biomass production was positively related to root length growth in *A. pseudoplatanus*, *F. excelsior*, *A. platanoides* and *T. cordata* as expected, while this relation was not significant in *C. betulus* and *F. sylvatica* (data not shown).

**Table 4:** Annual fine root length and surface area production (SA) per square meter ground area in 0–10 cm, 10–20 cm, 20–30 cm soil depth and for the profile (0–30 cm).

Species	Depth (cm)	Length (m m^-2^ year^-1^)	SA (cm^2^ m^-2^ year^-1^)
*Fraxinus excelsior*	0–10	1455 ± 479aA	17504 ± 5604aA
	10–20	1210 ± 231aA	14984 ± 2976aA
	20–30	638 ± 202bABC	8660 ± 2350bABC
	**Profile**	**3303 ± 639 A**	**41148 ± 7345 A**
*Acer pseudoplatanus*	0–10	903 ± 269aA	11617 ± 4004aAB
	10–20	340 ± 73aBC	3473 ± 945aB
	20–30	333 ± 72aABC	4376 ± 978aABC
	**Profile**	**1576 ± 300 B**	**19465 ± 4759 B**
*Acer platanoides*	0–10	907 ± 248aA	11030 ± 3140aAB
	10–20	933 ± 209aABC	10644 ± 2504aAB
	20–30	695 ± 188aAC	7584 ± 1927aAC
	**Profile**	**2535 ± 343 AB**	**29259 ± 4235 AB**
*Carpinus betulus*	0–10	1519 ± 567abA	13877 ± 4402aAB
	10–20	1089 ± 268aAC	10213 ± 2191aA
	20–30	553 ± 171bAB	5528 ± 2093bAB
	**Profile**	**3161 ± 796 A**	**29618 ± 6408 AB**
*Tilia cordata*	0–10	545 ± 179aA	6524 ± 2263aB
	10–20	615 ± 214aC	7665 ± 2666aAB
	20–30	275 ± 102aB	3918 ± 1331aA
	**Profile**	**1359 ± 395 B**	**17149 ± 5376 B**
*Fagus sylvatica*	0–10	560 ± 186aA	8036 ± 2109aAB
	10–20	808 ± 216abABC	10074 ± 2622abABC
	20–30	1095 ± 202bC	13589 ± 3052aC
	**Profile**	**2462 ± 417 AB**	**31699 ± 5106 B**

*Given are mean ± SE for eight plots. Statistically significant differences between the soil depths are indicated by different lower case letters, significant differences between the species in a soil depth by different capital letters; differences between the species in the 0–30 cm profile are indicated by bold capital letters (Mann–Whitney *U*-test; *p* < 0.05)*.

### Fine Root Dynamics in Relation to Root Properties and Aboveground Productivity

**FIGURE 5:**
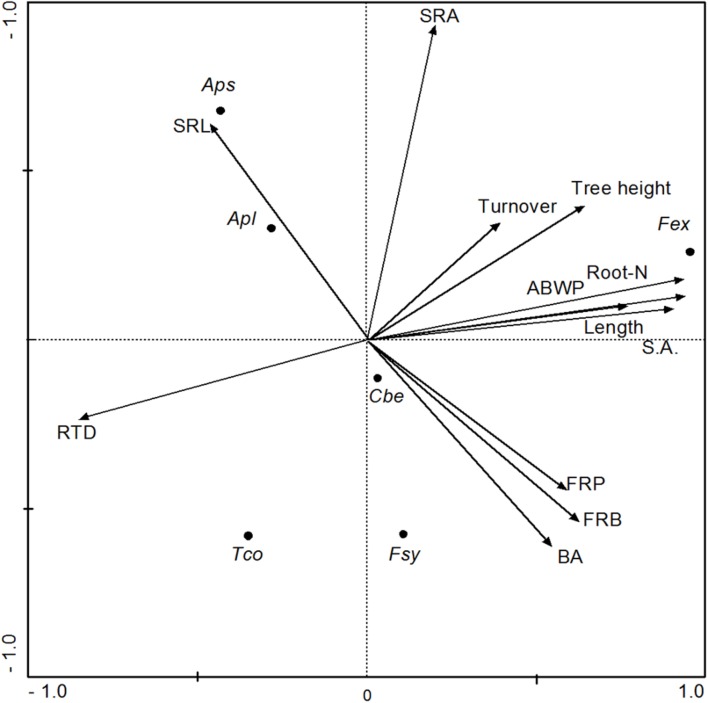
**Results of a Principal Components Analysis regarding the parameters fine root biomass (FRB), root morphological properties (RTD, SRA, SRL), annual production of fine root biomass (FRP), length (Length) and surface area (SA), root turnover, and tree basal area (BA) and aboveground woody biomass production (ABWP)**. Shown are the inter-relationships along the first two axes (axis 1 = x axis; axis 2 = y axis). Species: Fex, *Fraxinus excelsior*; Aps, *Acer pseudoplatanus*; Apl, *Acer platanoides*; Cbe, *Carpinus betulus*; Tco, *Tilia cordata*; Fsy, *Fagus sylvatica*.

To explore relationships between fine root morphology, FRP, and aboveground structure and productivity (ABWP) among the six tree species, a PCA with four axes was conducted. Fine root biomass production as well as fine root length and surface area growth were positively related to the first axis together with root nitrogen concentration and FRB. Aboveground woody biomass production and tree height were also positively associated with axis 1, while RTD was negatively related to this axis (**Table 5**). The SRA and SRL values of the produced fine root biomass in the ingrowth cores correlated closest with axis 2. Fine root turnover was the only variable not being associated with the other variables; it correlated with axis 3 (**Table 5**). The PCA plot in **Figure 5** indicates that the three EM species resemble each other in terms of root morphology and root productivity, while the two *Acer* species (AM) group separately, and *F. excelsior* (AM) seems to differ from the other five species in most tested influential parameters.

**Table 5:** Results of a principal components analysis (PCA) regarding the variables fine root biomass of the plots (FRB), root morphological properties, annual FRP and length and surface area production, fine root turnover, and aboveground tree structure, biomass and wood production (ABWP).

Variables	Axis 1	Axis 2	Axis 3	Axis 4
Eigenvalues	0.4809	0.2134	0.166	0.1023
FRP	**0.590**	-0.446	0.566	-0.359
Length production	**0.771**	0.099	0.555	-0.294
Surface area production	**0.900**	0.091	0.334	-0.121
Fine root turnover	0.398	0.351	**0.586**	**0.585**
FRB	**0.627**	-0.538	-0.344	-0.445
RTD	**-0.862**	-0.233	0.350	-0.006
SRA	0.202	**0.936**	-0.122	-0.260
SRL	-0.469	**0.644**	-0.111	-0.535
Root N concentration	**0.935**	0.181	-0.003	-0.011
BA of target species	0.542	-**0.614**	-0.499	0.108
Tree height	**0.645**	0.399	-0.607	0.137
ABWP	**0.939**	0.129	-0.151	0.270

*High interrelations are given in bold print*.

Contrary to expectation, species differences in FRP could not be explained by species differences in root morphological properties such as different mean fine root diameters, SRL, SRA, root tissue densities, or root N contents (**Table 6**). Neither root N concentration nor root tissue density correlated with FRP in any of the species (except for a negative relation between root N and FRP in *A. platanoides*). In *A. pseudoplatanus*, FRP was negatively related to SRA and SRL indicating that *A. platanoides* produced more fine root biomass, when the newly grown fine root branches were shorter and thicker. Similarly, species differences in root turnover could be explained neither by mean fine root diameter nor by other root morphological traits (Supplementary Table SI2).

**Table 6:** Spearman rank correlation coefficients (r_s_) for the relationship between aboveground productivity and morphological properties with FRP for the pooled data set (all six species) based on species means; ABWP, aboveground woody biomass production; SRL, specific root length; SRA, specific root area; RTD, root tissue density; MD, mean diameter; root N, fine root nitrogen concentration.

	*r*_s_	*p*
ABWP	0.371	0.469
SRL	-0.257	0.623
SRA	-0.314	0.544
RTD	-0.371	0.469
MD	-0.143	0.787
Root N	0.600	0.208

*None of the relationships were significant at *p* < 0.05*.

Aboveground productivity (ABWP) was not related to FRP in the 0–30 cm profile, neither in the sample of all species (**Table 6**) nor in separate correlation analyses at the species level, except for *C. betulus* (**Table 7**). Relating ABWP to root traits across the six species revealed a significant negative relation to root tissue density; this relation disappeared, when *F. excelsior* with particularly thick and N-rich fine roots was excluded (data not shown). The species means of ABWP were not related to the species SRL, SRA, or root N concentration means (Supplementary Table SI3). When this analysis was conducted at the species level, none of the ABWP – root trait relations were significant at *p* < 0.05 (in *F. sylvatica*, a marginally significant relation at 0.05 < *p* < 0.1 between ABWP and root N appeared; Supplementary Table SI4).

**Table 7:** Spearman rank correlation coefficients (r_s_) for the relationship between aboveground productivity and morphological properties with FRP conducted separately for the six species.

	Species					
	*Fraxinus excelsior*	*Acer pseudoplatanus*	*Acer platanoides*	*Carpinus betulus*	*Tilia cordata*	*Fagus sylvatica*
ABWP	0.024	0.190	-0.476	0.881^∗^	0.429	-0.262
SRL	-0.167	-0.929^∗^	-0.190	-0.143	-0.595	-0.524
SRA	-0.286	-0.810^∗^	-0.238	-0.071	-0.524	-0.524
RTD	0.571	0.333	0.048	0.238	-0.238	0.429
MD	-0.429	0.738^∗^	0.048	0.119	0.524	0.167
Root N	0.429	0.143	-0.491	-0.381	-0.167	-0.357

*Significant correlations at *p* < 0.05 are marked with an asterisk. ABWP, aboveground woody biomass production; SRL, specific root length; SRA, specific root area; RTD, root tissue density; root N, fine root N concentration*.

## Discussion

### Factors Influencing Fine Root Longevity

We obtained mean fine root turnover rates between 0.16 and 1.60 year^-1^ in the six species and three horizons for the bulked fine root biomass <2 mm, equivalent to a mean root lifespan of 0.6 year (*A. platanoides*) to 6.3 years (*F. excelsior*); this is a 10-fold difference between the species. The majority of turnover figures, however, ranged between 0.3 and 0.7 year^-1^ (i.e., lifespans of 1.4–3.3 years). With minirhizotron observation, Withington et al. (2006) found median fine root lifespans between 0.6 and 2.5 years for *A. pseudoplatanus, A. platanoides, T. cordata* and *F. sylvatica*, which matches well with our ingrowth core-based estimates, given that Withington et al. (2006) considered only first-order roots, while our data include also the higher root orders with longer life spans in the bulked root mass <2 mm diameter, and Withington et al. (2006) did not investigate *F. excelsior*. In agreement with Withington et al. (2006), we found a relatively high lifespan in *A. pseudoplatanus*, while our data indicate a short mean lifespan of *A. platanoides* roots, in contrast to their results. It has to be kept in mind that the results of minirhizotron and ingrowth core studies on fine root turnover in forests are often poorly comparable (Burke and Raynal, 1994), and turnover rates derived from minirhizotron observation typically are higher than ingrowth core estimates (e.g., Finér et al., 2011). This offers another explanation of the higher lifespan values found in our study compared to the figures of Withington et al. (2006). Further, the trees in the common garden study of Withington et al. (2006) likely were exposed to lower root competition intensity than the trees in the mixed forest of our study, which could also have influenced root longevity. Finally, climatic differences between the studies of Withington et al. (2006) and our study (Poland vs. Germany) could partly explain differences in longevity values (cf. Leppälammi-Kujansuua et al., 2014).

Comparing fine root turnover among different species based on the bulked <2 mm fine root biomass instead of focusing on root orders might introduce errors, if the species differ largely in their branching patterns and the functionality of the respective segments is different (McCormack et al., 2015). Earlier root order-based morphological analyses of the six species by Kubisch et al. (2015) showed that the branching patterns of the species were relatively similar despite belonging to different families and mycorrhiza types. For example, the fine root mass <2 mm in diameter of all species consisted to about 95% of the root orders 1–4, while higher order-segments contributed always with less than 5% to fine root biomass. Further, mean diameter, fine root length fraction and root tissue density as important morphological traits influencing nutrient acquisition followed in all species a remarkably similar trend from the first to the fourth order, suggesting that root segments with either absorptive functions or storage and conductance functions occupied rather similar proportions of fine root biomass in these species. McCormack et al. (2015) assume that the fourth root order should in temperate tree species mainly be responsible for nutrient and water transport, while nutrient and water acquisition is located in the first to third root orders. Even though we do not have information on the longevity of individual root segments and orders, we assume that the observed species differences in fine root turnover of the <2 mm-class should not result from different branching patterns and contrasting proportions of first- and second-order segments in the species, but rather reflect species differences in overall fine root longevity, as the species were relatively similar with respect to fine root length fractions. Clearly, a detailed root order-based analysis of root lifespan would give a more reliable picture of species differences in the dynamic properties of the fine root system and of possible contrasts between EM and AM species.

We could not detect relationships between root longevity and root morphological and chemical properties in our six-species sample. This is unexpected and does contrast with the findings of McCormack et al. (2012, 2015) who reported a positive relation between root lifespan and fine root diameter, root C/N ratio and root Ca concentration, and a negative one between lifespan and SRL for North-American tree species. However, analysis of covariance showed for our data a positive effect of SRL on fine root turnover, when separated from the influence of mycorrhiza type. This is a hint that thinner roots were shorter lived, even though mean fine root diameter was not an influential factor in our analysis. In a literature review of fine root lifespan in temperate tree species, Guo et al. (2008a) found that lifespan generally increased with root diameter. Similarly, Eissenstat et al. (2015) measured a higher median fine root lifespan in thick-rooted AM tree species. These results refer to the first two root orders. For grasses, Ryser (1996) reported higher root longevity when root tissue density was higher, while a relation to diameter did not appear. These observations indicate that root production is indeed behaving in a manner, which fits to the resource optimization concept proposed by Eissenstat and Yanai (1997). Large-diameter roots require higher investment of carbon and nutrients per unit root length or surface area, which should be coupled with greater root lifespan in order to ensure a favorable nutrient and water return on the amount of carbon and nutrients invested.

In our sample, mean fine root diameter differed only moderately among the six species (means of 0.33–0.59 mm) and greater root lifespan was found not only in the species with largest diameters (*F. excelsior* and *T. cordata*) but also in *A. pseudoplatanus* with the thinnest fine roots. Large spatial and also temporal variation in fine root turnover (McCormack et al., 2014) together with only limited species differences in root diameter may explain our failure to detect relationships between fine root morphology and lifespan in this mixed forest. The species sample of Eissenstat et al. (2015) covered a much greater range of root diameters (0.2–1.3 mm) and referred only to AM species.

### Are Fine Root Productivity and Root Lifespan Different between EM and AM Tree Species?

In contradiction to our first hypothesis, the three EM tree species of our sample differed not significantly from the three AM species in terms of FRP and fine root turnover when comparing the group means (see **Table 3**). The two groups were also similar with respect to the belowground: aboveground production ratio. We had expected that *F. excelsior* and the two *Acer* species as AM species would have longer-lived fine roots because fine root longevity has been found to increase with root diameter (McCormack et al., 2012), and *F. excelsior* had the thickest fine roots of the six species. Further, the minirhizotron data of Withington et al. (2006) indicate that the two *Acer* species have particularly long fine root lifespans (median lifespan of first and second order roots: 1.5 and 2.6 years in *A. pseudoplatanus* and *A. platanoides*, respectively). This result was only in part confirmed by our ingrowth core study which showed a very high longevity of the *A. pseudoplatanus* roots (means of 2.3–4.8 years for roots <2 mm in diameter), but not of the *A. platanoides* roots (0.6–0.9 year). In fact, mean fine root diameter was not different between AM and EM species in our sample, and *A. pseudoplatanus* had particularly thin roots in the first four root orders (Kubisch et al., 2015). The morphological comparison showed that systematic differences between the two mycorrhiza types did only exist with respect to SRA (higher values in AM species), but not for root diameter, RTD, SRL, or root N concentration in our sample. In an analysis of 25 North-American woody species, Comas and Eissenstat (2009) reported for the EM species a higher branching intensity (number of root tips per total root length) than for the AM species; this was not visible in our six-species sample (Kubisch et al., 2015).

The long lifespan of the two *Acer* species observed by Withington et al. (2006) was explained with a very thick exodermis in the fine roots of the two *Acer* species. Our results with the diverging *Acer* species suggest that this explanation may not be generally valid.

A shortcoming of our approach is that the root morphological and productivity measurements refer to the standard size class of fine roots <2 mm in diameter in all species, thus including a substantial fraction of non-mycorrhizal root mass in the analysis. This could have masked potential effects of mycorrhizal type on root morphology and dynamics. Experimental duration may also have influenced the results. For example, Ostonen et al. (2005) observed different contributions of roots < 1 mm and roots > 1 mm diameter to FRP over the course of a 3 years-long root production study.

Despite these possible sources of bias, it appears that the type of mycorrhiza is a less important factor influencing fine root lifespan than other possibly relevant factors. Previous research has shown that plant-internal resource allocation rules (Eissenstat and Duncan, 1992) and external abiotic and biotic factors act as the main determinants of fine root lifespan, among the latter nutrient availability, drought stress, temperature extremes and the activity of root herbivores, pathogens and fungal symbionts (Wells and Eissenstat, 2002; Guo et al., 2008b; Rasmann and Agrawal, 2008; Adams and Eissenstat, 2015). Most of the abiotic factors should have been more or less similar among the study plots of the six species in Hainich forest, while differences in herbivore and pathogen activity may vary with the specific chemical and biological conditions in the rhizosphere of the species (Guckland et al., 2009; Cesarz et al., 2013; Scheibe et al., 2015).

Interesting is the direct comparison of fine root dynamics in the two coexisting congeners *A. pseudoplatanus* and *A. platanoides*, which may reveal the development of different strategies of belowground resource foraging in closely related tree species. The two congeners showed marked morphological differences (more tips per root mass, a higher fine root surface area and thinner second and fourth order root segments in *A. pseudoplatanus* than in *A. platanoides*) and a higher overall fine root biomass in *A. pseudoplatanus*. Thus, it appears that tree species with similar fine root diameters as in the Hainich mixed forest can achieve elevated resource uptake rates either through maintaining a large surface area of first-order roots (*A. pseudoplatanus*) or by frequently turning over the existing fine root mass (*A. platanoides*) which should increase mean root uptake capacity by reducing mean root age (Eissenstat and Yanai, 1997). In our species sample, species differences in root tip frequency (tips per root mass) were the most influential fine root morphological traits (Kubisch et al., 2015).

In tree species assemblages with higher phylogenetic diversity as in tropical or subtropical moist forests, fine root morphology often is more variable among different species than in the temperate mixed forest of our study. Under these conditions, nutrient foraging strategies may largely depend on fine root diameter, with thin-root species often showing greater fine root growth rates, whereas thick-root species are apparently relying more on mycorrhizal fungi with respect to nutrient acquisition. Across 14 evergreen or deciduous broad-leaf or coniferous AM trees in subtropical China, Liu et al. (2015) found much larger fine root diameter variation (0.19–0.86 mm) than in our study (0.33–0.59 mm), which was associated with differences in root growth rate and the degree of AM colonization.

### Aboveground – Belowground Linkages

While leaf lifespan was more or less similar among the six species (6–7 months), the lifespan of fine roots (averaged over all fine root mass <2 mm) varied up to fivefold among the species (ca. 11–54 months) and up to threefold between the horizons within a species. This suggests that fine root and leaf lifespan are only poorly related to each other in this species sample, and fine root longevity is controlled by other factors than aboveground phenology. A similar conclusion was drawn by Withington et al. (2006) for their five-species broadleaf tree sample, which included four of our species. In a global literature survey, Finér et al. (2011) detected no significant influence of stand basal area or stem density on fine root turnover. We also found no relation between the species’ aboveground woody biomass production and FRP and root turnover, disproving our third hypothesis. However, across all species, wood production increased with mean fine root diameter and decreased with increased root tissue density. We speculate that, in a fertile soil with more or less stable nutrient-rich patches as in Hainich forest, species with thicker fine roots may achieve a greater nutrient return on resource investment in root mass than thin-root species; this could promote aboveground productivity.

### Relative Importance of First- and Second-Order Roots in Fine Root Dynamics

The detailed root order-related biomass analysis showed that about 30–50% of total fine root biomass (<2 mm) referred to the first and second orders which are assumed to conduct most of nutrient and water uptake (Guo et al., 2008b) and are termed absorptive roots (McCormack et al., 2015). These segments contain the fine root tips and the directly adjacent finest root segments with generally lowest degree of suberization. Assessment of the resource foraging strategies of the six species should therefore primarily consider the amount of carbon invested into the production of first- and second-order roots. We multiplied total FRP as measured in the ingrowth cores with the biomass fractions of the first to fourth root orders as found in the FRB inventory of Kubisch et al. (2015), assuming similar root longevity in the different root orders. Extrapolating the biomass distribution key from the inventory to the ingrowth cores may be justified in our study, because we found similar branching patterns of the fine root strands in the inventory and the ingrowth core analysis for all six species (**Figure 1**). According to this very rough calculation, the species produced between 50 and 90 g root biomass per m^2^ and year in 0–30 cm soil depth in the first and second root orders, which is much less than the global FRP mean (337 g m^-2^ year^-1^, mean sampling depth 37 cm, total root biomass <2 mm) given by Finér et al. (2011) for temperate forests. To our knowledge, there are only two other studies that attempted to quantify the root order-related root production on a plot level basis (Xia et al., 2010; *Fraxinus mandshurica*; Sun et al., 2011: *Fraxinus mandshurica* and *Larix gmelinii*, both roughly 25 years-old plantations). No other study seems to exist, where such an approach has been applied to mature (>100 years-old) forest stands. In these two East Asian tree plantations, much lower FRP values [42 and 27 g m^-2^ year^-1^ in 0–10 cm soil depth (Xia et al., 2010) or 0–20 cm soil depth (Sun et al., 2011)] were reported than in our mature stands, even though the same methodology (ingrowth cores) was used at least in the latter study. Given that temperate trees invest about 300 g m^-2^ year^-1^ in leaf biomass, and coarse and large root production has its equivalent in twig and branch production, we draw the conclusion that temperate trees achieve nutrient and water uptake at lower cost for absorbing structures than is needed for carbon assimilation. Nevertheless, the total length of absorbing roots produced annually in the topsoil is enormous, exceeding 1 km per m^2^ ground area in the 0–30 cm profile in the six species investigated here.

## Conclusion

The co-existence of EM and AM tree species in cool-temperate mixed forests raises the question about possible differences in belowground resource foraging strategies between these two tree groups. In our sample of six relatively wide-spread species, variation in root dynamics occurred mainly within the two groups and not between them, contradicting our main hypothesis. Investigation of a larger number of tree species might reveal significant group differences in fine root lifespan and root productivity, but there exist only few other AM tree species in Central Europe with wider distribution (e.g., *Acer campestre* and *Ulmus glabra*). Since many of the common species were included in our sample, our results and those of Kubisch et al. (2015) on root morphological differences suggest that species differences in fine root morphology, lifespan, and growth rate in Central European broadleaved mixed forests are primarily determined by species identity, while the influence of mycorrhiza type is only of secondary importance. Species differences manifested primarily in differences in root tip frequency, while variation in root diameter was of minor importance (Kubisch et al., 2015).

Possible differences between ECM and AM species in root morphology and turnover in many cases will be overlain by effects of environmental and stand structural variation (Finér et al., 2011; Leppälammi-Kujansuua et al., 2014). In correspondence, a global analysis of measured fine root turnover rates in forests (Finér et al., 2011) seems to suggest that temperature (or growing season length), and not mycorrhiza type, is a main determinant of tree fine root lifespan and FRP, because mean turnover rate continuously increased from 0.77 to 1.21 and 1.44 year^-1^ and FRP from 311 to 428 and 596 g m^-2^ year^-1^ from boreal to temperate and tropical forests. Thus, a root productivity increase and root lifespan decrease is occurring with both biome transitions, i.e., from the boreal to the temperate forest and from the temperate to the tropical forest, even though a shift in predominant mycorrhiza type (from EM to AM) does occur only between temperate and tropical forests, but not between boreal and (cool) temperate forests. More data from species-rich mixed forests is needed to understand the influence of mycorrhiza type on tree fine root morphology, dynamics, and functioning.

## Author Contributions

Study idea and design: CL and DH; field and lab work: PK; data analysis: PK and DH; paper concept and writing: PK, DH, and CL.

## Conflict of Interest Statement

The authors declare that the research was conducted in the absence of any commercial or financial relationships that could be construed as a potential conflict of interest.
