# Supplementary material for: Fine Root Productivity and Turnover of Ectomycorrhizal and Arbuscular Mycorrhizal Tree Species in a Temperate Broad-Leaved Mixed Forest
Source: Front Plant Sci. 2016 Aug 26;7:1233. doi: 10.3389/fpls.2016.01233 (PMC5000521; doi:10.3389/fpls.2016.01233)
Supplement: Supplementary file 1 [file Table_1.PDF]

Table SI 1. Fine root biomass (in g m^{-2}) in the six plot types at the beginning of the study in June 2011 (only fine root biomass of target species). Significant differences ($p < 0.05$) between the species within a soil depth are marked with different capital letters.

		Soil depth		
Species	0-10 cm	10-20 cm	20-30 cm	Profile
Fraxinus excelsior	113.0 ± 26.2 B	109.7 ± 51.2 AB	47.5 ± 15.7ABC	270.2 ± 17.5 AB
Acer pseudoplatanus	74.5 ± 12.4 AB	66.8 ± 17.8 A	53.1 ± 14.7 B	194.3 ± 8.5 AB
Acer platanoides	59.5 ± 21.1 AB	53.1 ± 11.8 A	29.1 ± 15.3 A	141.6 ± 9.5 A
Carpinus betulus	103.5 ± 29.0 AB	87.4 ± 17.1AB	55.9 ± 15.9 ABC	246.8 ± 12.6 AB
Tilia cordata	51.5 ± 25.1 A	93.3 ± 28.3 AB	73.1 ± 16.4 BC	217.9 ± 13.6 AB
Fagus sylvatica	55.2 ± 27.2 A	142.0 ± 27.8 B	103.4 ± 30.2 C	300.6 ± 17.4 B
