# Supplementary material for: Fine Root Productivity and Turnover of Ectomycorrhizal and Arbuscular Mycorrhizal Tree Species in a Temperate Broad-Leaved Mixed Forest
Source: Front Plant Sci. 2016 Aug 26;7:1233. doi: 10.3389/fpls.2016.01233 (PMC5000521; doi:10.3389/fpls.2016.01233)
Supplement: Supplementary file 2 [file Table_2.PDF]

Table SI 2. Pearson correlation coefficients (r) for the relation between fine root turnover (unit: yr^{-1}) and five root traits based on species means. None of the relationships was significant at $p < 0.05$. FRP = fine root productivity, ABWP = aboveground woody biomass production.

	r	*p*
SRL	-0.29	0.29
SRA	0.18	0.37
RTD	-0.17	0.37
MD	-0.04	0.46
Root N	0.37	0.24
FRP	0.21	0.35
ABWP	0.50	0.16
