# Supplementary material for: Fine Root Productivity and Turnover of Ectomycorrhizal and Arbuscular Mycorrhizal Tree Species in a Temperate Broad-Leaved Mixed Forest
Source: Front Plant Sci. 2016 Aug 26;7:1233. doi: 10.3389/fpls.2016.01233 (PMC5000521; doi:10.3389/fpls.2016.01233)
Supplement: Supplementary file 3 [file Table_3.PDF]

Table SI 3. Pearson correlation coefficients (r) and probability of error (p) for the relationship between aboveground woody biomass production (ABWP) and fine root traits in the sample of six species based on species means. Significant relations ($p < 0.05$) are printed in bold.

	r	*p*
SRL	-0.464	0.178
SRA	0.258	0.313
RTD	**-0.870**	**0.013**
MD	**0.535**	**0.012**
Root N	0.763	0.137
