# Supplementary material for: Fine Root Productivity and Turnover of Ectomycorrhizal and Arbuscular Mycorrhizal Tree Species in a Temperate Broad-Leaved Mixed Forest
Source: Front Plant Sci. 2016 Aug 26;7:1233. doi: 10.3389/fpls.2016.01233 (PMC5000521; doi:10.3389/fpls.2016.01233)
Supplement: Supplementary file 4 [file Table_4.PDF]

Table SI 4. Pearson correlation coefficients (r) for the relation between aboveground woody biomass production (ABWP, in g m^{-2} yr^{-1}) and five root traits in the six species. None of the relationships was significant at $p < 0.05$; relationships with $0.05 < p < 0.01$ are marked with ° SRL= specific root length; SRA= specific root area; RTD= root tissue density; Root N= root nitrogen concentration.

	Species					
	Fraxinus excelsior	*Acer pseudoplatanus*	*Acer platanoides*	*Carpinus betulus*	*Tilia cordata*	*Fagus sylvatica*
SRL	-0.254	0.223	-0.044	-0.269	-0.225	0.186
SRA	-0.247	-0.117	0.312	-0.133	-0.231	0.498
RTD	0.215	-0.021	-0.436	0.119	-0.028	-0.614
MD	0.160	0.429	0.268	0.456	0.377	0.451
Root N	0.088	0.021	-0.033	-0.519	-0.358	0.662°
